# In‐Source Collision‐Induced Dissociation (CID) Improves Higher‐Energy Collisional Dissociation (HCD)‐Dependent Fragmentation of ADP‐Ribosyl Peptides

**DOI:** 10.1002/rcm.9961

**Published:** 2024-12-04

**Authors:** Taku Kasai, Yuto Nakamura, Masanori Aikawa, Sasha A. Singh

**Affiliations:** ^1^ Department of Medicine, Center for Interdisciplinary Cardiovascular Sciences, Division of Cardiovascular Medicine, Brigham Women's Hospital Harvard Medical School Boston Massachusetts USA; ^2^ Division of Cardiovascular Medicine, Center for Excellence in Vascular Biology, Brigham and Women's Hospital Harvard Medical School Boston Massachusetts USA; ^3^ Department of Medicine, Channing Division of Network Medicine, Brigham Women's Hospital Harvard Medical School Boston Massachusetts USA

**Keywords:** ADP‐ribosylation, electron transfer dissociation, in‐source collision‐induced dissociation, ribosylome

## Abstract

**Rationale:**

ADP‐ribosylation is a posttranslational modification whose higher‐energy collisional dissociation (HCD) products are dominated by complete or partial modification losses, complicating peptide sequencing and acceptor site localization efforts. We tested whether in‐source collision‐induced dissociation (CID) performed on a quadrupole‐Orbitrap could convert ADPr to the smaller phosphoribose‐H_2_O derivative to facilitate HCD‐dependent peptide sequencing.

**Methods:**

ADP‐ribosyl (ADPr) peptides derived from the human macrophage‐like cell line THP‐1 were analyzed on a quadrupole‐Orbitrap. We monitored the dissociation of ADPr (+ 541.061 Da) to phosphoribosyl‐H_2_O (+ 193.997 Da) peptides while varying the source and high‐field asymmetric waveform ion mobility mass spectrometry (FAIMS) compensation voltages. Xcorr and ptmRS were used to evaluate peptide sequencing and acceptor site confidence, respectively. Phosphoribosyl‐H_2_O acceptor sites were compared with those determined by electron‐transfer higher‐energy collision dissociation (EThcD), performed on a quadrupole‐ion trap‐Orbitrap.

**Results:**

In‐source CID of ADPr peptides to their phosphoribosyl‐H_2_O derivatives increased with increasing source voltage (up to 50 V), as judged by monitoring the corresponding modification loss ([adenosine monophosphate/AMP]^+^) and the number of identified phosphoribosyl‐H_2_O peptide identifications. The average Xcorr increased from 1.36 (ADPr) to 2.26 (phosphoribosyl‐H_2_O), similar to that achieved with EThcD for ADPr peptides (2.29). The number of high‐confidence acceptor sites (> 95%) also increased, from 31% (ADPr) to 70% (phosphoribosyl‐H_2_O), which was comparable to EThcD (70%).

**Conclusions:**

In‐source CID converts ADP‐ribosyl to phosphoribosyl‐H_2_O peptides that are more amenable to HCD‐dependent peptide sequencing, providing an alternative method for acceptor site determination when ETD‐based methods are not available.

## 1 Introduction

Adenosine diphosphate ribosylation (ADP‐ribosylation) is a posttranslational modification involving the transfer of the ADP‐ribosyl (ADPr) moiety of NAD to proteins; a reaction catalyzed by protein ADP‐ribosyltransferases (PARPs) [1, 2]. Mammalians encode 17 PARPs, some of which catalyze poly‐ADP‐ribosylation (PAR), but most catalyze mono‐ADP‐ribosylation (MAR) [3]. Our interest in ADP‐ribosylation began when we discovered that the pro‐inflammatory cytokine, interferon‐γ, induced the transcription and translation of PARP14 and PARP9 in macrophages [4]. At the time, mass spectrometry‐based workflows to study ADP‐ribosylation were just emerging [5, 6, 7], but timely as we implemented them in our own research [8].

Specifically, we adopted the ADP‐ribosylome workflow by the Nielsen and Hottiger labs in which MARylated peptides are analyzed: using poly‐ADPr‐glycohydrolase (PARG), PARylated peptides are enzymatically hydrolyzed to their MARylated forms since only the monomer is amenable to mass spectrometric analysis [5, 6, 7]. Several studies, including our own, have leveraged the pros of various dissociation methods to improve ADPr peptide sequencing and/or amino acid acceptor site localization, namely, higher‐energy collisional dissociation (HCD) and electron transfer dissociation (ETD)‐based methods [9, 10, 11, 12].

**FIGURE 1:**
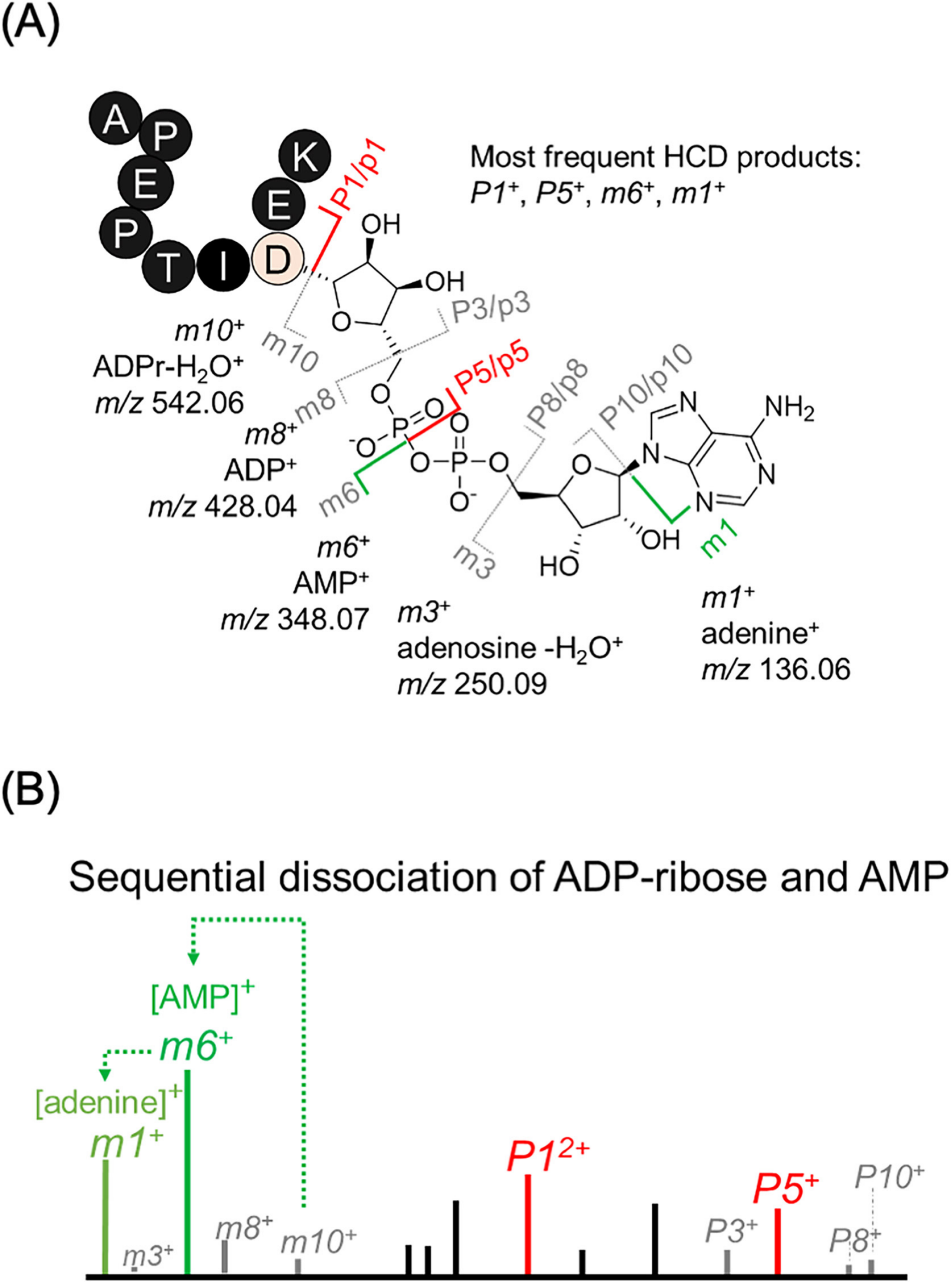
The dissociation properties of adenosine diphosphate ribose (ADPr). (A) HCD‐dependent fragment ions of ADPr peptides. “P” indicates intact peptide after the modification (*m*‐ion) loss; “p” indicates sequential dissociation of the peptide backbone. The *P5*‐*m6* fragment ions are the targets of this study. (B) A schematic of summarizing that ADPr *m*‐ions result from the sequential dissociation of ADPr or AMP (Kuraoka et al., 2021).

When using HCD, the ADP‐ribose is labile producing complex products that are a combination of complete and partial modification losses, forming the *m*‐ions and *P*‐ions (Figure 1A) [13]. The *m*‐ions ([*m1*/adenine]^+^, [*m3*/adenosine‐H_2_O]^+^, [*m6*/adenine monophosphate]^+^, [*m8*/adenine diphosphate]^+^, [*m10*/ADPr]^+^) are used as diagnostic ions for ADPr peptide‐specific spectra [11]. We developed an ADPr peptide MS/MS annotation tool, RiboMaP, that enabled us to understand ADPr peptides' dissociation properties, by monitoring the frequency of the *m*‐ions and *P*‐ions as a function of dissociation method, collision energy, or instrument platform [11, 14]. We demonstrated that despite the presence of most *m*‐ions in HCD spectra, they do not necessarily form from direct dissociation of the ADPr peptide, but instead, from sequential dissociation of the larger *m*‐ions into the smaller *m*‐ions (Figure 1B) [11]. We also established that the *m10*/*P1* and *m6*/*P5* dissociation products are the most vulnerable dissociation events, and that sequential dissociation of the *P5*‐ion (precursor peptide with a phosphoribose‐H_2_O, referred to herein as phosphoribose*) into *b*‐ or *y*‐ions still harboring the partial modification (*p5*‐ions) can be used for acceptor site localization [11, 14, 15].

Although our laboratory has prioritized HCD‐based characterization of ADPr peptides, we have also employed EThcD to confirm acceptor sites [8, 11]. Nonetheless, in recognition that not all labs have access to ETD capability, we tested the concept that nozzle‐skimmer dissociation also known as, in‐source collision‐induced dissociation (CID) [16, 17, 18, 19, 20], could be used to pre‐fragment ADPr peptides into the *P5* peptide precursors that are better suited for HCD. The use of in‐source CID as an alternative to or a supplement of a dedicated dissociation method dates back decades. In‐source CID can be used on single quadrupole instruments when a dedicated collision cell is not available [20, 21]. In‐source CID has also been used to screen for glycopeptides by generating their low *m/z* diagnostic ions [18], and to pre‐fragment large peptide/proteins to facilitate subsequent dissociation steps [22, 23, 24]. These applications inspired us to investigate the potential for in‐source CID‐HCD to improve ADPr peptide annotation by converting the relatively labile ADPr modification to a more stable, smaller footprint, the phosphoribosyl* (or *P5* ion) moiety. Moreover, we implemented this in‐source CID‐HCD strategy when using gas‐phase fractionation, including high‐field asymmetric waveform ion mobility mass spectrometry (FAIMS), to increase the signal‐to‐noise of the ADPr peptides above non‐ADPr peptide contaminants [15].

## 2 Methods

### 2.1 mzCloud Compound Data

The HCD curves for adenosine diphosphate ribose (ADPr), adenosine diphosphate (ADP), and adenosine monophosphate (AMP) were acquired from the mzCloud database (https://www.mzcloud.org/). The compound IDs are as follows: ADPr (1771, negative ion mode; Orbitrap Elite), ADP (20 066, positive ion mode, Orbitrap ID‐X), and AMP (19 810, positive ion mode, Orbitrap ID‐X).

### 2.2 Cell Culture

THP‐1 cells, a human monocytic cell line derived from an acute monocytic leukaemia patient, were purchased from American Type Culture Collection (Cat# TIB‐202) and maintained in Roswell Park Memorial Institute (RPMI) 1640 medium (Fisher Scientific, Cat# MT10040CV) in 10% fetal bovine serum (VWR International, Cat# 97068‐085) with 1% penicillin and streptomycin (Thermo Fisher Scientific, Cat# 15140163) at 37 °C in 5% CO_2_. THP‐1 cells were plated at a density of 3.0 × 10^7^ cells in 15 cm dishes and allowed to differentiate from their monocyte‐like state into macrophages using RPMI supplemented with 100 ng/mL phorbol 12‐myristate 13‐acetate (PMA, Sigma‐Aldrich, Cat# P1585) for 2 days, followed by a media exchange back to RPMI alone for 1 day. The cells were washed twice with ice‐cold PBS, and then the cells were lysed in modified RIPA buffer (50 mM Tris–HCl pH 7.4, 0.4 M NaCl [Sigma‐Aldrich, Cat# S9888], 1.0 mM EDTA [Boston Bio Products, Cat# BM‐150], 1.0% nonidet P‐40 [Sigma‐Aldrich, Cat# 74385], 0.1% sodium deoxycholate [Sigma‐Aldrich, Cat# D6750], 40 μM PJ34 [Abcam, Cat# ab120981], 1.0 μM ADP‐HPD [Millipore Sigma, Cat# 118415], protease inhibitor cocktail [Sigma‐Aldrich, Cat# P8340]) as described previously [8].

### 2.3 Proteolysis

THP‐1 lysates were homogenized on ice using sonication. Homogenized lysates were precipitated in acetone (Fisher Scientific, Cat# A949‐1). The cell pellets were resuspended in a denaturation buffer (6.0 M urea [Sigma‐Aldrich, Cat# U4884], 2.0 M thiourea [Sigma‐Aldrich, Cat# T7875], 10 mM HEPES [Boston BioProducts, Cat# BBH‐80]). The protein amount was determined by a Pierce 660 nm Protein Assay Reagent (Thermo Fischer Scientific, Cat# 22660). Proteins (5.0–10 mg) were reduced in 1.0 mM dithiothreitol (DTT, Thermo Fisher Scientific, Cat# 20290) and alkylated in 5.5 mM chloroacetamide (Sigma‐Aldrich, Cat# C0267). Proteolysis was performed with trypsin (Thermo Fisher Scientific, Cat# 90058) in 20 mM ammonium bicarbonate (Sigma‐Aldrich, Cat# 09830) overnight. The peptides were desalted using Sep‐Pak C18 Classic Cartridge (Waters, Cat# WAT051910) by following the manufacturer's instructions. Using a Concentrator plus complete system (Eppendorf AG), the peptide sample was reduced to a final volume of 0.8 mL of affinity precipitation buffer (50 mM Tris–HCl pH 7.4, 10 mM MgCl_2_ [Thermo Fisher Scientific, Cat# AM9530G], 250 μM DTT, 50 mM NaCl). The peptide amount was determined by using a NanoDrop One Spectrophotometer at 205 nm (ε_205_ = 31 methods, Thermo Fisher Scientific).

### 2.4 ADPr Peptide Enrichment

The peptides (2–4 mg) were treated with PARG overnight (1.0 μg PARG per 1.0 mg peptide, Creative BioMart, Cat# PARG‐31H) to obtain only MARylated peptides [25]. The peptides were enriched using the *Archaeoglobus fulgidus* ADP‐ribosylation binding eAf1521 macrodomain affinity pull‐down strategy, as described previously [11, 26]. The enriched ADPr peptides were processed with a molecular weight cutoff filtration step using the Microcon‐30 kDa Centrifugal Filter Unit (Millipore Sigma, Cat# MRCF0R03). The peptides were desalted using Oasis HLB cartridges (30 mg [Waters, 1 cc, Cat# WAT094225] for the MARylated peptides) by following the manufacturer's instructions and resuspended in loading buffer (5.0% acetonitrile [Fisher Scientific, Cat# A955‐1], 0.5% formic acid [Fisher Scientific, Cat# 28905] in water [Fisher Scientific, Cat# W6‐1]) for mass spectrometric analysis.

### 2.5 Mass Spectrometry

ADPr peptides were analyzed on the Exploris 480 fronted with a FAIMS Pro [15] and EASY‐Spray Source, coupled to an Easy‐nLC1200 HPLC pump (Thermo Fisher Scientific); and the Orbitrap Fusion Lumos fronted with EASY‐Spray Source, coupled to an Easy‐nLC1000 HPLC pump (Thermo Fisher Scientific). The peptides were fractionated with a trap‐elute column set‐up: an Acclaim PepMap 100 C18 HPLC Columns, 75 μm × 70 mm (Thermo Fisher Scientific, Cat# 164946); and an EASY‐Spray HPLC Column, 75 μm × 250 mm (Thermo Fisher Scientific, Cat# ES902). Binary mobile phases (A:water/0.1% formic acid; B: 95% acetonitrile/5% water/0.1% formic acid) were employed at the flow rate of 300 nL/min. The analytical gradient was from 5% to 21% B for 60 min, followed by 10 min of 21% to 30% B, and a 15‐min column wash alternating between 95% B and 5% B, held for 3 min each, in between a 2‐min ramp up or down. Post the washes, the column was equilibrated at 95% B. The mass spectrometers were operated in positive mode.

#### 2.5.1 In‐Source CID Properties of ADPr Peptides

Each *m*‐ion (Figure 1A) which is *m/z* 136.062 (adenine^+^, *m1*), 250.093 (adenosine‐H_2_O^+^, *m3*), 348.069 (AMP^+^, *m6*), 428.035 (ADP^+^, *m8*), 524.057 (ADPr‐2H_2_O^+^, *m10*) and 542.067 (ADPr‐H_2_O^+^, *m10*) was monitored while increasing the source voltage from 0 to 60 V in 4 V increments divided into four MS1‐only acquisitions (*m/z* 130–550; separate injections): injection 1‐ 0, 4, 8, and 12 V; injection 2‐ 16, 20, 24, and 28 V; injection 3‐ 32, 36, 40, and 44 V; and injection 4‐ 48, 52, 56, and 60 V. The MS1 resolution was set to 120 000. The *m*‐ion intensities were calculated using the area under the curve of the monoisotopic peak's extracted ion chromatogram of the entire analytical gradient.

#### 2.5.2 In‐Source CID‐HCD Properties of ADPr Peptides

The MS1 resolution was set to 120 000 with a scan range of *m/z* 400–1200. Ions with a charge state of 2–6 were selected for MS2 fragmentation, dynamic exclusion was auto, and the intensity threshold was set to 50 000. The total cycle time of the data‐dependent mode was set to 3 s. The MS2 resolution set to 120 000, isolation window was 2.0 *m/z*, maximum injection time was auto, and normalized HCD collision energy was 26%. The in‐source CID voltages of 26, 28, 30, 32, 34, and 48 V were applied. Gas‐phase segmentation (GPS) is defined as segmenting the MS1 scan range, analyzed as separate injections: *m/z* 400–705 and *m/z* 695–1000 when analyzing ADPr peptides; and *m/z* 400–605, *m/z* 595–805, and *m/z* 795–1200 when analyzing phosphoribosyl* peptides. FAIMS was operated on the standard resolution set to static gas mode with a total carrier gas flow of 3.9 L/min. Single compensation voltages (CVs) of −40, −50, and −60, or multiple CVs of −40 V/−50 V/−60 V or −60 V/−70 V/−80 V were applied.

#### 2.5.3 EThcD Properties of ADPr Peptides

The MS1 resolution was set to 120 000 with a scan range of *m/z* 400–1500 (Orbitrap Fusion Lumos). Ions with a charge state of 2–6 were selected for MS2 fragmentation, dynamic exclusion was auto, and intensity threshold was set to 25 000. The total cycle time of the data dependent mode was set to 3 s. The MS2 resolution was set to 120 000, isolation window was 1.2 *m/z*, maximum injection time was dynamic, and supplemental activation collision energy was 22.5%.

### 2.6 Spectral Annotation

ADPr peptide spectra were analyzed using Proteome Discoverer 2.4 (Thermo Fisher Scientific) custom‐built with the ADPr spectral annotation module, RiboMaP [11, 14] or Proteome Discoverer 2.5. The spectra were queried against the Uniprot human fasta database (downloaded 18 January 2022; *n* = 100 730 entries) using the SEQUEST search engine algorithm. Trypsin was set as the digestion enzyme, allowing up to 4 missed cleavages and a minimum peptide length of 6 amino acids. ADPr (+ 541.061 Da) or phosphoribose* (+ 193.997 Da) of Asp, Glu, Lys, Arg, Ser, Thr, Tyr and His; oxidation (+ 15.995 Da) of methionine; and acetylation (+ 42.011 Da) of the N‐terminus, were set as variable modifications. Carbamidomethylation (+ 57.021 Da) of cysteine was set as a static modification. Spectral mass tolerances were 10 ppm for the precursor and 20 mmu for the fragment ions. The peptide false discovery rate (FDR) was calculated using Percolator (target/decoy method) and peptide spectrum matches (PSMs) were filtered at 1.0% FDR. The ADPr peptide spectra were also annotated and scored for the *m*‐series and *p*‐series, fragment ions unique to ADPr peptides [11, 14]. Proteome Discoverer 2.5 using ptmRS was used for calculating ADPr and phosphoribosyl* site probabilities. A summary of all data acquired in this study is provided in Table S1.

### 2.7 Data Representation

The PSMs, peptide groups, and protein groups for each dataset were exported from Proteome Discoverer. Data plots were done using Microsoft Excel from Microsoft 365.

### 2.8 Data Availability Statement

The mass spectrometry proteomics data have been deposited to the ProteomeXchange Consortium via the PRIDE [12] partner repository with the dataset identifier PXD055586 and 10.6019/PXD055586.

## 3 Results and Discussion

### 3.1 Study Concepts

**FIGURE 2:**
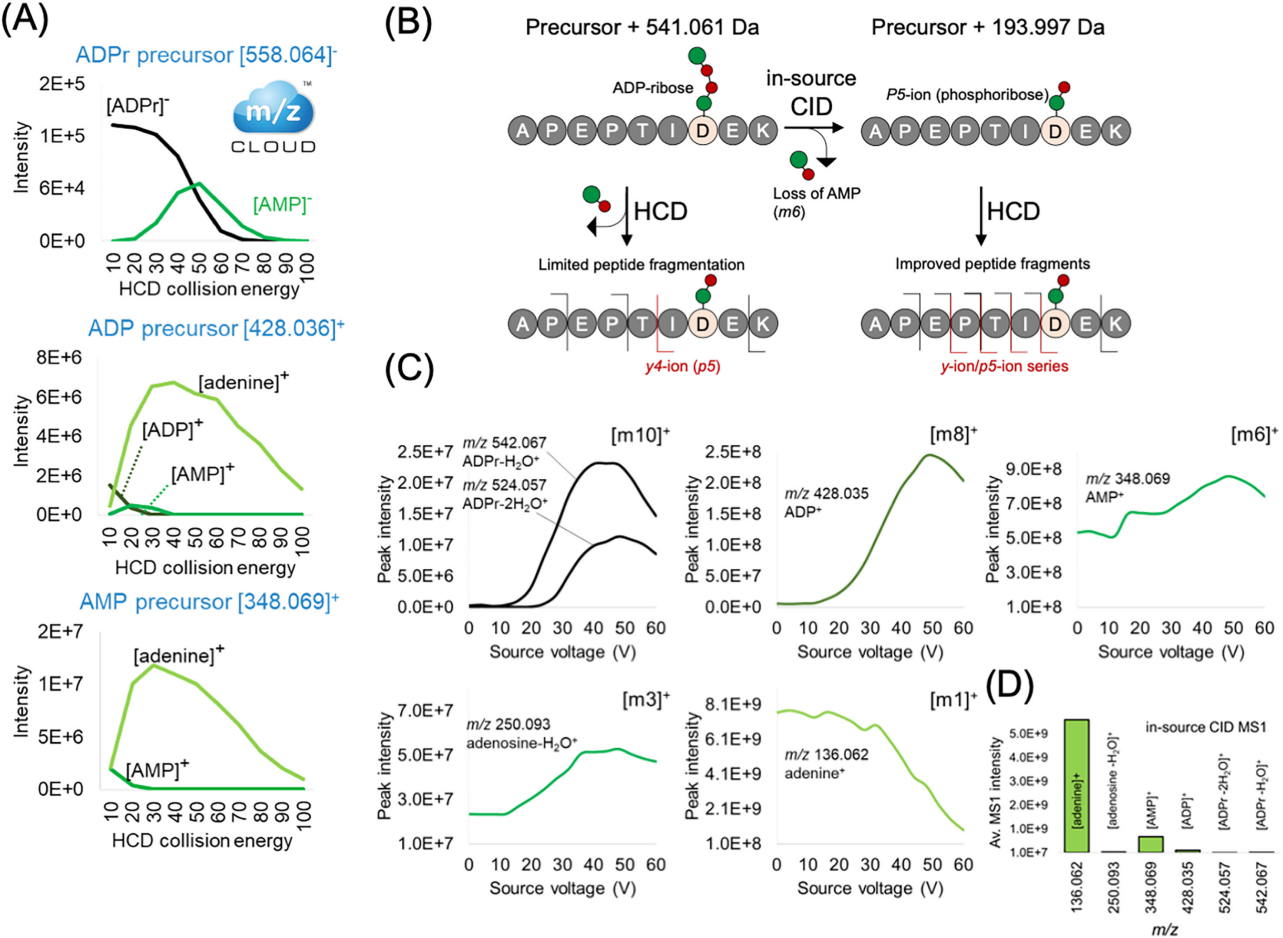
Rationale for in‐source CID of ADPr peptides. (A) Dissociation curves for ADPr, ADP, and AMP small molecules (source: mzCloud), demonstrating their precursor to product relationship in HCD. (B) Strategy and hypothesized fragmentation results for in‐source CID‐HCD of ADPr peptides on the benchtop quadrupole‐Orbitrap (Exploris 480) mass spectrometer. (C) MS1 scans (*m/z* 130–550) of ADPr peptides enriched from THP‐1 cells acquired with increasing source voltage. Peak intensity was calculated using the monoisotopic peak extracted over a 70‐min gradient. (D) The average MS1 intensity (across source voltages) for each ADPr ion.

Our previous study [11] demonstrated that ADPr (*m10*) and AMP (*m6*) are the major collision‐induced dissociation modification losses for ADPr peptides (Figure 1A). We also demonstrated that the lower mass *m*‐ions result from the sequential dissociation of ADPr or AMP rather than from the ADPr peptide itself [11]. Mass spectrometric analysis of ADPr, ADP (*m8*), and AMP small molecules (Figure 2A, source:mzCloud) underscore this precursor‐to‐product relationship (notably, ADPr → AMP → adenine) that accounts for the dominance of the [AMP]^+^ and [adenine]^+^ ions typical of ADPr peptide MS2 spectra [14].

Moreover, the *m6*/*P5* dissociation provides a phosphoribose* footprint that is more stable than the ADPr counterpart during the dissociation of the peptide backbone itself: *P5*‐ion (precursor ion) to *p5*‐ion (fragment ion) transition [11]. The phosphoribose* increases the chance of identifying the amino acid acceptor site (Figure 2B). We therefore rationalized that in‐source CID of ADPr peptides could provide *P5*‐ion/phosphoribose* precursors for subsequent HCD, that in turn would improve peptide fragmentation and acceptor site localization (Figure 2B).

### 3.2 In‐Source CID Properties of ADPr Peptides

The goal for in‐source CID is to promote the *m6*/*P5* dissociation event, while limiting over‐ and non‐specific fragmentation events. We monitored the *m*‐ions at distinct source voltages and quantified their signals in MS1‐only scans (*m/z* 130–550). All *m*‐ions but the *m1*‐ion increased in intensity until their signals peaked at around 40 V, whereas the *m1*‐ion/[adenine]^+^ peaked between 0 and 4 V demonstrating that even without an applied voltage, baseline in‐source CID occurs (Figure 2C). Similar to ADPr peptides' MS2 spectra [11], the [adenine]^+^ and [AMP]^+^ ions exhibit the strongest signals in positive ion mode (Figure 2D). Based on these curves, we determined that a source voltage less than 50 V would be suitable to generate phosphoribosyl* peptide precursors, without over‐fragmentating the ADPr peptides.

### 3.3 The Impact of FAIMS on Phosphoribosyl* Peptide (*P5*‐Ion) Precursor Properties

**FIGURE 3:**
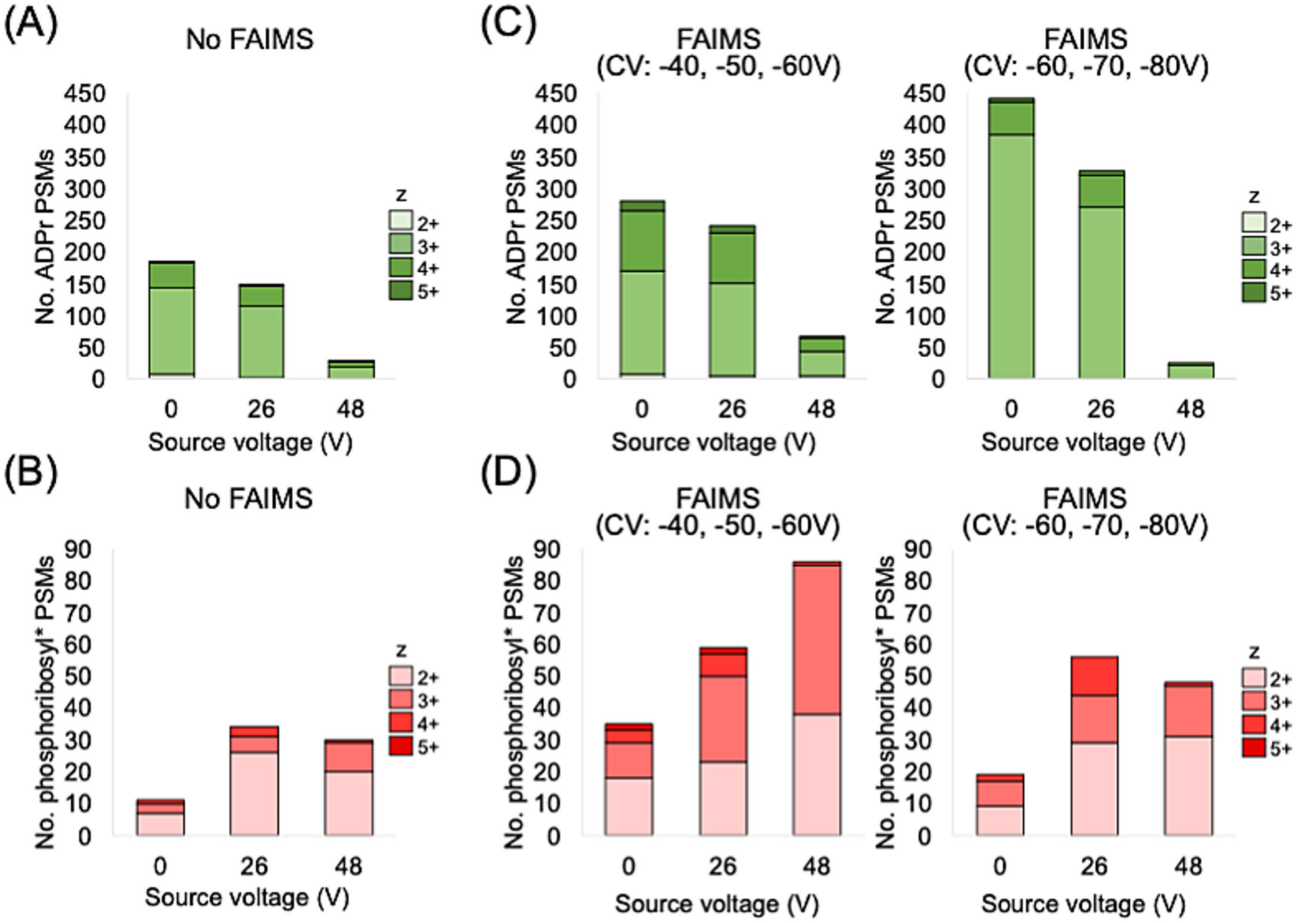
An evaluation of source voltage and FAIMS on ADPr and phosphoribosyl* peptide yields. (A,C) The number of ADPr peptides identified at three test source voltages with or without FAIMS. (B, D) The number of phosphoribosyl* peptides identified at three test source voltages with or without FAIMS. *, —H_2_O.

We first tested three source voltages (0, 26, and 48 V) and evaluated ADPr versus phosphoribosyl* peptide yields with or without FAIMS. In a standard acquisition (without FAIMS), we confirmed that the number of ADPr PSMs decreased with increasing source voltage, whereas the number of phosphoribosyl* PSMs increased confirming that the source promoted the *m6*/*P5* dissociation event (Figure 3A,B).

In our previous study, we determined that high negative compensation voltages (i.e., −60 to −90 V) stabilize ADPr peptides, owing to their relatively high charge states (z = 3^+^ prominent) compared with non‐ADPr peptide contaminants (z = 2^+^ prominent) that are filtered out [15]. In this study, we applied two independent FAIMS strategies to each in‐source CID experiment. As expected, both compensation voltage configurations (−40 V/−50 V/−60 V and −60 V/−70 V/−80 V) increased the number of ADPr PSMs up to two‐fold more than the standard acquisition (Figure 3C). In each case, an increasing source voltage decreased the number of ADPr PSMs, providing the corresponding increase in the number of phosphoribosyl* PSMs (Figure 3D). It is interesting to note that the phosphoribosyl* peptides shift to a z = 2^+^ prominence which is expected with the [m6]^+^ modification loss. The lower negative multiple compensation voltages therefore preserved phosphoribosyl* peptide.

### 3.4 In‐Source CID‐Generated Phosphoribosyl* Precursors Improve ADPr Peptide Sequencing

We next refined the in‐source CID conditions, but in lieu of monitoring the *m*‐ions we instead monitored the yields of ADPr and phosphoribosyl* peptides analyzed between 26 and 34 V. In this optimization, one injection was analyzed per source voltage, corresponding to the suitable FAIMS compensation voltage settings (−40 V/−50 V/−60 V) for phosphoribosyl* peptides described in Figure 3.

**FIGURE 4:**
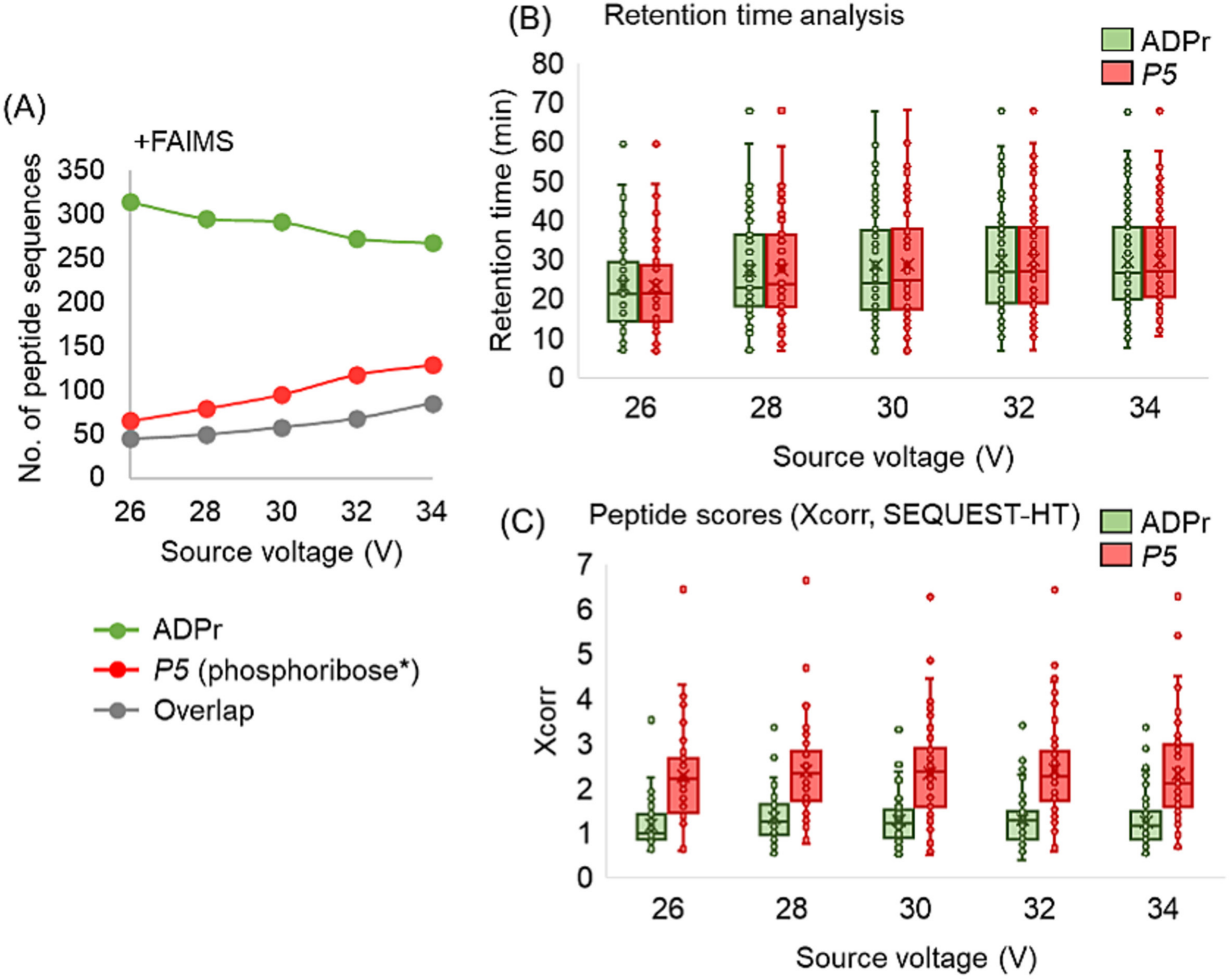
Further in‐source CID optimization with FAIMS. (A) The number of unique ADPr vs. phosphoribosyl* peptide (*P5*) sequences at increasing source voltage. Overlap, common peptides sequences (inclusive to the ADPr and *P5* plots). (B) Retention time (RT) distributions for common peptides. Source‐induced *P5* dissociation products will have identical RTs as their ADPr peptide precursors. (C) Peptide scores (Xcorr values from SEQUEST) are higher for the *P5* precursors demonstrating improved *b*/*y* dissociation with the smaller modification (phosphoribosyl* vs. ADPr). *, —H_2_O.

A source voltage of 34 V provided the greatest number of phosphoribosyl* peptide identifications, for example, 65 at 26 V versus 129 at 34 V (Figure 4A). Due to the dissociation of ADPr to phosphoribosyl* precursors, the number of ADPr peptide identifications decreased under the same conditions (315 at 26 V vs. 268 at 34 V), (Figure 4A). Some modified peptides were common to the ADPr and phosphoribosyl* peptide precursors (85 at 34 V). We confirmed that these common peptides have the same retention times (Figure 4B). Additionally, the phosphoribosyl* peptide scores (Xcorr, SEQUEST‐HT) are higher (av. of all in‐source CID conditions = 2.36) compared with their ADPr counterparts (av = 1.28), supporting our initial hypothesis that in‐source CID of ADPr to phosphoribosyl* peptides would improve amino acid sequencing (Figure 2B).

### 3.5 In‐Source CID‐Generated Phosphoribosyl* Precursors Improve Acceptor Site Localization

With improved peptide sequencing, we also expected that the phosphoribosyl* would allow us to increase the number of high‐confidence acceptor sites. Our previous works, by focusing on HCD‐dependent sequencing, prioritized ADPr peptide yields rather than acceptor site localization. When necessary, we relied on the EThcD performed on the Orbitrap Fusion Lumos to confirm acceptor sites [8, 11].

In this study, we also analyzed the ADPr peptide samples using EThcD to corroborate acceptor sites identified with the phosphoribosyl* precursor. In this specific results section, ADPr peptides labeled as HCD‐derived were from the in‐source CID‐HCD experiment, to keep track of the ADPr to phosphoribosyl* peptide conversions and compare the overlapping modified peptides.

**FIGURE 5:**
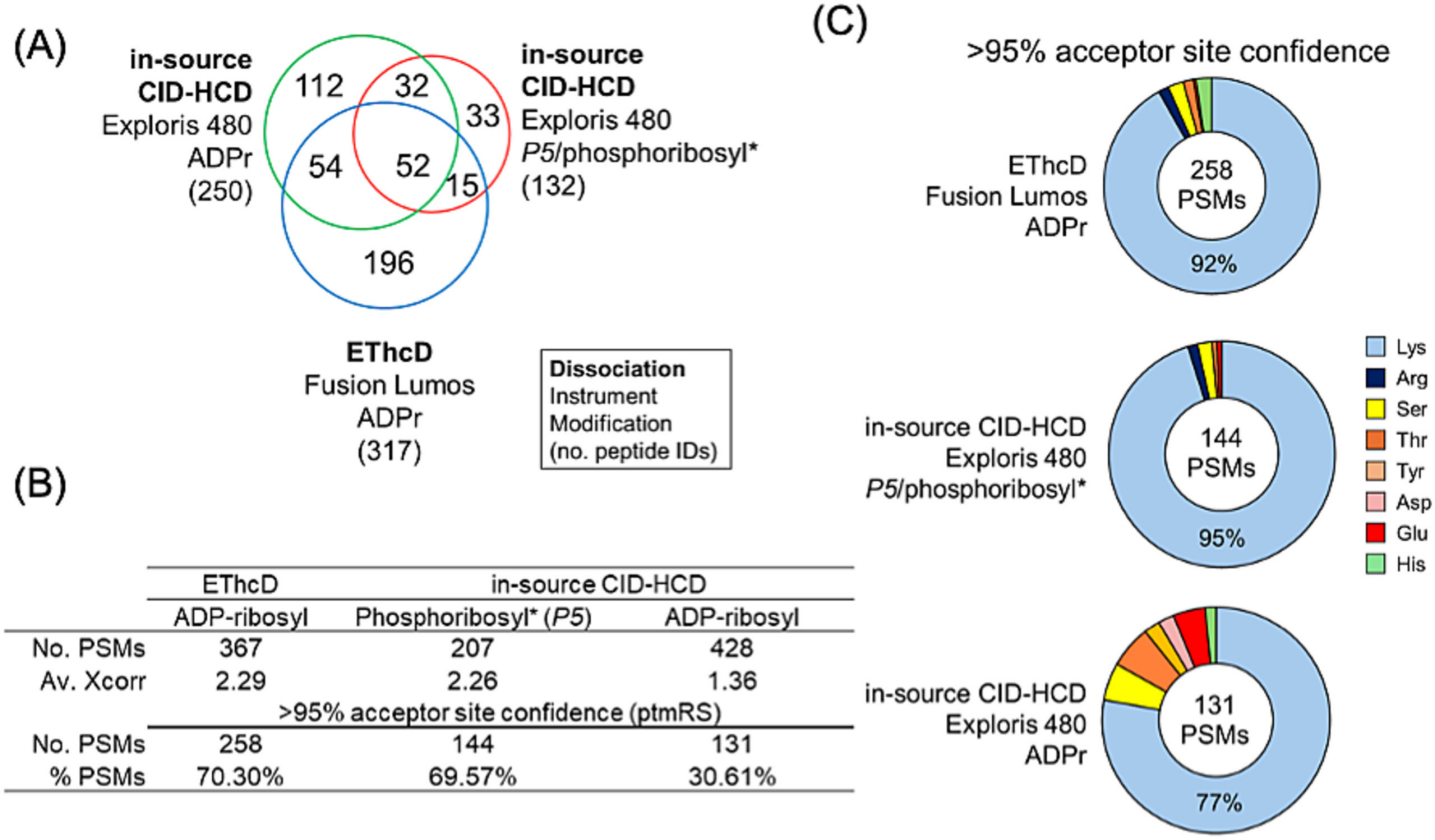
In‐source CID‐HCD‐generated phosphoribosyl* precursors improve ADPr peptide sequence quality. (A) Venn diagram displaying extent of overlapping sequence identifications across the acquisition strategies. (B) A summary of Xcorr and acceptor site probabilities across the acquisition strategies. (C) Distribution of amino acid acceptor sites with > 95% localization confidence (ptmRS in Proteome Discoverer). *, —H_2_O.

**FIGURE 6:**
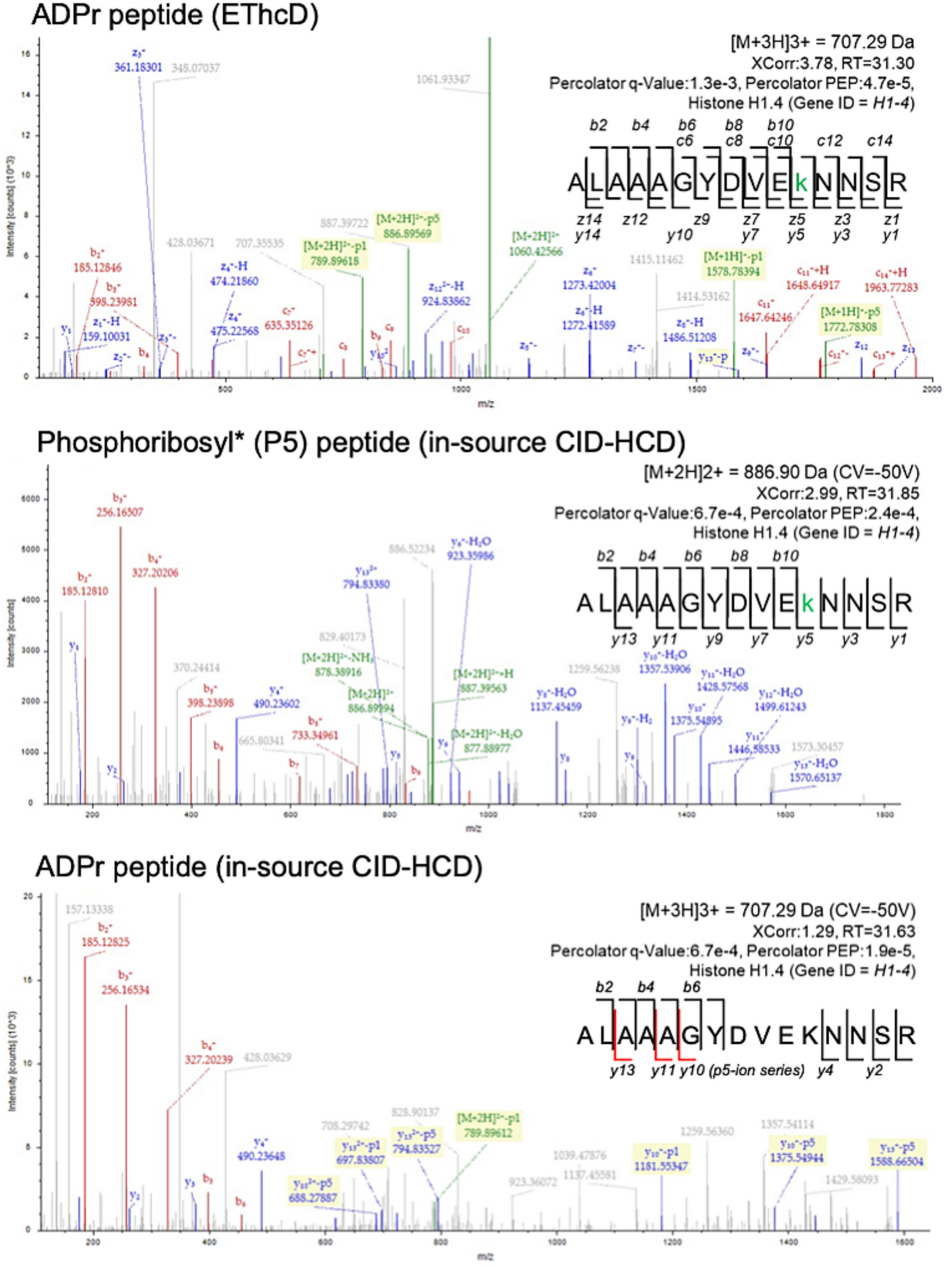
An example ADPr peptide identified as its ADPr and phosphoribosyl* forms using either EThcD performed on a Fusion Lumos or in‐source CID‐HCD performed on an Exploris 480. Both EThcD of the ADPr form, and in‐source CID‐HCD of the phosphoribosyl* form confirmed the acceptor site to be the lysine (y5); however, when analyzed by HCD, upstream Asp and Glu are also candidate acceptor sites. *, —H_2_O. Highlighted peaks are ADPr peptide fragment ions annotated using RiboMaP.

We identified 250 and 317 ADPr peptides with in‐source CID‐HCD and EThcD, respectively; and 132 phosphoribosyl* peptides with in‐source CID‐HCD (Figure 5A, Table S2). A closer look at their average Xcorr values demonstrated that ADPr PSMs analyzed with EThcD and phosphoribosyl* PSMs analyzed with in‐source CID‐HCD gave comparable scores (2.29 and 2.26, respectively); whereas ADPr PSMs analyzed with HCD alone had the lowest average score (1.36) (Figure 5B, Table S2). The number of high‐confidence acceptor site PSMs was also comparable between EThcD (ADPr) and in‐source CID‐HCD (phosphoribosyl*)—70% of modified PSMs with > 95% confidence (ptmRS)—a marked improvement over HCD (ADPr, 31% PSMs with > 95% confidence). Although all three acquisition strategies support lysine as the most frequent acceptor site, the proportion of high‐confidence lysines is > 90% for EThcD (ADPr) and in‐source CID‐HCD (phosphoribosyl*), as compared with 77% for HCD (ADPr) (Figure 5B,C, Tables S2). Figure 6 provides an example commonly identified modified peptide (ALAAAGYDVEKNNSR, Histone H1.4) whose acceptor site (Lys‐11) is conclusive with EThcD (ADPr) and in‐source CID‐HCD (phosphoribosyl*) spectra, but indistinguishable amongst Asp‐8, Glu‐10 and Lys‐11 when relying on HCD (ADPr) alone. In‐source CID of ADPr to the phosphoribosyl* peptide form therefore improves on the number of high‐confidence amino acceptor site identifications when compared with HCD alone.

### 3.6 Implementing in‐Source CID‐HCD to Maximize the Number of ADPr Peptides and Acceptor Sites

**FIGURE 7:**
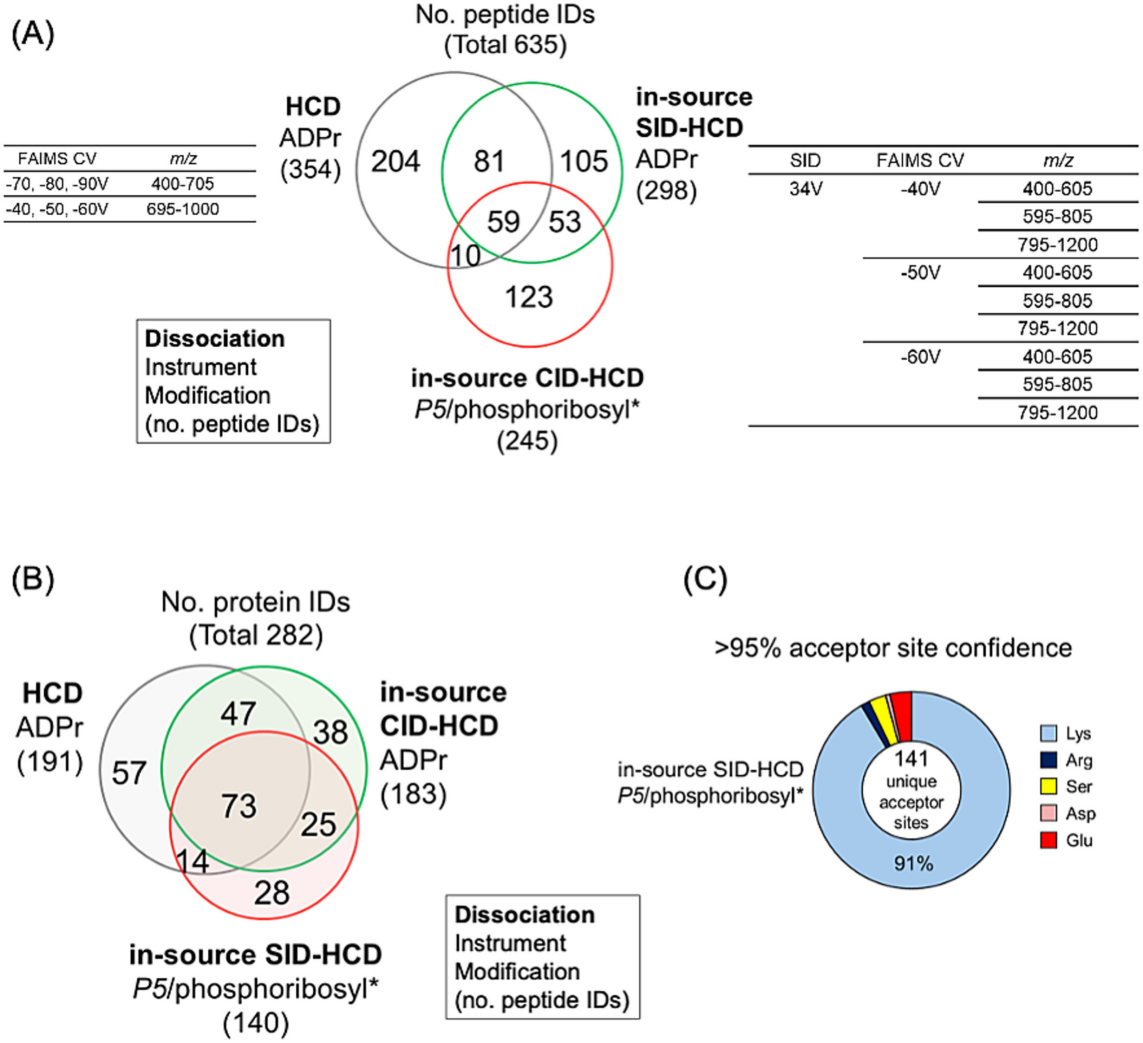
In‐source CID‐HCD with GPS + FAIMS provides the maximum number of *P5*/phosphoribosyl* peptide. (A) Venn diagram displaying extent of overlapping ADPr and *P5*/phosphoribosyl* peptide and (B) proteins. *, —H_2_O. Inset (A) the number of unique acceptor sites identified using the phosphoribosyl* precursor. (C) The number and distribution of acceptor sites identified in the THP‐1 ADP‐ribosylome when capitalizing on in‐source CID‐HCD‐derived phosphoribosyl* precursors.

Our final analysis presents a typical acquisition strategy for ADPr peptides analyzed on the quadrupole‐Orbitrap. To maximize the number of ADPr peptide identifications, GPS with FAIMS operated at multiple compensation voltages (CVs −70/−80/−90 V for *m/z* 400–705, CVs −40/−50/−60 V for *m/z* 695–1000) provides the maximum number of ADPr peptide identifications (Figure 7A, Table S3) [15].

To then maximize the number of phosphoribosyl* forms, we re‐analyzed the samples using three GPS scans (*m/z* 400–605, *m/z* 595–805, and *m/z* 795–1200) at three distinct single FAIMS compensation voltages (−40, −50, and −60 V) with an in‐source CID voltage of 34 V (Figure 7A Table S4). This in‐source CID‐HCD analysis provided redundant and new ADPr peptide identifications which is expected since the latter is operated at lower negative compensation voltages that stabilize distinct ADPr peptides [15]. Combined we identified 512 ADPr peptides corresponding to 254 proteins (Figure 7B, Tables S5 and S6).

The in‐source CID‐HCD then provided 245 phosphoribosyl* forms that overlap with 122 of the 512 ADPr peptides, with an additional unique 123 phosphoribosyl* peptides, for a combined modified 635 peptides and 282 modified proteins, respectively (Figure 7A,B, Table S7). Overlapping ADPr and phosphoribosyl* peptides provide corroborating sequence identifications. ADPr peptide identifications enabled by unique phosphoribosyl* peptide identifications are an additional benefit of the in‐source CID‐HCD strategy. In this final analysis, 141 unique modification acceptor sites of which 91% are lysine were identified (Figure 7C, Table S8); a statistic that we would not have been able to report when conducting traditional HCD‐dependent ADP‐ribosylomics.

## 4 Conclusions

Our study confirmed that the in‐source CID‐induced smaller phosphoribose* precursor for HCD improved backbone fragmentation and ADPr acceptor site localization. Although not investigated in this study, it may be feasible to generate specific phosphoribose* precursors using in‐source CID in combination with targeted mass spectrometry (e.g., parallel reaction monitoring, selected reaction monitoring), for ADPr peptides that were not immediately identified with the in‐source CID‐HCD discovery mode. In conclusion, in‐source CID‐HCD is an alternative method for ADPr acceptor site determination when ETD is not available.

## Author Contributions

**Taku Kasai:** conceptualization, investigation, writing–original draft, methodology, validation, visualization, formal analysis, data curation. **Yuto Nakamura:** investigation, methodology, writing–review and editing. **Masanori Aikawa:** conceptualization, funding acquisition, writing–review and editing, project administration, supervision. **Sasha A. Singh:** conceptualization, investigation, writing–original draft, methodology, validation, visualization, formal analysis, project administration, supervision.

## Disclosure

TK and YN are employees of Kowa Company, Ltd., Nagoya, Japan, but also current visiting scientists at Brigham and Women's Hospital and Harvard Medical School when the study was conducted.

## Supporting information


**Table S1.** Datasets acquired in this study.
**Table S2.** Peptide spectrum matches' data for the ADPr and phosphoribosyl* peptides with EThcD and in‐source CID‐HCD (filtered by high confidence and search engine (SEQUEST‐HT) rank 1).*, ‐H_2_O.
**Table S3.** Peptide groups' data for the ADPr peptides using HCD with FAIMS + GPS at 2 injections (filtered by high confidence and search engine rank 1 at PSMs).
**Table S4.** Peptide groups' data for the ADPr and phosphoribosyl* peptides using in‐source CID‐HCD with FAIMS + GPS at 9 injections (filtered by high confidence and search engine rank 1 at PSMs).*, ‐H_2_O.
**Table S5.** Proteins' data for the ADPr peptides using HCD with FAIMS + GPS at 2 injections (filtered by high confidence and search engine rank 1 at PSMs).
**Table S6.** Proteins' data for the ADPr peptides using in‐source CID‐HCD with FAIMS + GPS at 9 injections (filtered by high confidence and search engine rank 1 at PSMs).
**Table S7.** Proteins' data for the phosphoribosyl* peptides using in‐source CID‐HCD with FAIMS + GPS at 9 injections (filtered by high confidence and search engine rank 1 at PSMs). *, ‐H_2_O.
**Table S8.** The acceptor sites identified in the THP‐1 ADP‐ribosylome when capitalizing on in‐source CID‐HCD‐derived phosphoribosyl* precursors (filtered by high confidence and search engine rank 1 at PSMs, > 95% acceptor site confidence). *, ‐H_2_O.

## References

[1] W. L. Kraus , “PARPs and ADP‐Ribosylation: 60 Years on,” Genes & Development 34 (2020): 251–253. 32040441 10.1101/gad.336420.120 PMC7050492

[2] T. Honjo , Y. Nishizuka , and O. Hayaishi , “Diphtheria Toxin‐Dependent Adenosine Diphosphate Ribosylation of Aminoacyl Transferase II and Inhibition of Protein Synthesis,” Journal of Biological Chemistry 243, no. 12 (1968): 3553–3555. 4297784

[3] V. Schreiber , F. Dantzer , J. C. Ame , and G. de Murcia , “Poly (ADP‐Ribose): Novel Functions for an Old Molecule,” Nature Reviews. Molecular Cell Biology 7, no. 7 (2006): 517–528. 16829982 10.1038/nrm1963

[4] H. Iwata , C. Goettsch , A. Sharma , et al., “PARP9 and PARP14 Cross‐Regulate Macrophage Activation via STAT1 ADP‐Ribosylation,” Nature Communications 7 (2016): 12849. 10.1038/ncomms12849 PMC5095532 27796300

[5] S. Jungmichel , F. Rosenthal , M. Altmeyer , J. Lukas , M. O. Hottiger , and M. L. Nielsen , “Proteome‐Wide Identification of Poly (ADP‐Ribosyl)ation Targets in Different Genotoxic Stress Responses,” Molecular Cell 52, no. 2 (2013): 272–285. 24055347 10.1016/j.molcel.2013.08.026

[6] S. C. Larsen , M. Leutert , V. Bilan , et al., “Proteome‐Wide Identification of in Vivo ADP‐Ribose Acceptor Sites by Liquid Chromatography‐Tandem Mass Spectrometry,” Methods in Molecular Biology 1608 (2017): 149–162. 28695509 10.1007/978-1-4939-6993-7_11

[7] R. Martello , M. Leutert , S. Jungmichel , et al., “Proteome‐Wide Identification of the Endogenous ADP‐Ribosylome of Mammalian Cells and Tissue,” Nature Communications 7 (2016): 12917. 10.1038/ncomms12917 PMC5056437 27686526

[8] H. Higashi , T. Maejima , L. H. Lee , et al., “A Study Into the ADP‐Ribosylome of IFN‐Gamma‐Stimulated THP‐1 Human Macrophage‐Like Cells Identifies ARTD8/PARP14 and ARTD9/PARP9 ADP‐Ribosylation,” Journal of Proteome Research 18, no. 4 (2019): 1607–1622. 30848916 10.1021/acs.jproteome.8b00895 PMC6456868

[9] S. C. Buch‐Larsen , I. A. Hendriks , J. M. Lodge , et al., “Mapping Physiological ADP‐Ribosylation Using Activated Ion Electron Transfer Dissociation,” Cell Reports 32, no. 12 (2020): 108176. 32966781 10.1016/j.celrep.2020.108176 PMC7508052

[10] B. M. Zee and B. A. Garcia , “Electron Transfer Dissociation Facilitates Sequencing of Adenosine Diphosphate‐Ribosylated Peptides,” Analytical Chemistry 82, no. 1 (2010): 28–31. 19928949 10.1021/ac902134y

[11] S. Kuraoka , H. Higashi , Y. Yanagihara , et al., “A Novel Spectral Annotation Strategy Streamlines Reporting of Mono‐ADP‐Ribosylated Peptides Derived From Mouse Liver and Spleen in Response to IFN‐Gamma,” Molecular & Cellular Proteomics 21, no. 4 (2022): 100153. 34592425 10.1016/j.mcpro.2021.100153 PMC9014395

[12] V. Bilan , M. Leutert , P. Nanni , C. Panse , and M. O. Hottiger , “Combining Higher‐Energy Collision Dissociation and Electron‐Transfer/Higher‐Energy Collision Dissociation Fragmentation in a Product‐Dependent Manner Confidently Assigns Proteomewide ADP‐Ribose Acceptor Sites,” Analytical Chemistry 89, no. 3 (2017): 1523–1530. 28035797 10.1021/acs.analchem.6b03365

[13] S. M. Hengel , S. A. Shaffer , B. L. Nunn , and D. R. Goodlett , “Tandem Mass Spectrometry Investigation of ADP‐Ribosylated Kemptide,” Journal of the American Society for Mass Spectrometry 20, no. 3 (2009): 477–483. 19070509 10.1016/j.jasms.2008.10.025 PMC3073872

[14] S. A. Singh , S. Kuraoka , D. V. S. Pestana , W. Nasir , B. Delanghe , and M. Aikawa , “The RiboMaP Spectral Annotation Method Applied to Various ADP‐Ribosylome Studies Including INF‐Gamma‐Stimulated Human Cells and Mouse Tissues,” Frontiers in Cardiovascular Medicine 9 (2022): 851351. 35419443 10.3389/fcvm.2022.851351 PMC8996112

[15] T. Kasai , S. Kuraoka , H. Higashi , B. Delanghe , M. Aikawa , and S. A. Singh , “A Combined Gas‐Phase Separation Strategy for ADP‐Ribosylated Peptides,” Journal of the American Society for Mass Spectrometry 34, no. 10 (2023): 2136–2145. 37589412 10.1021/jasms.3c00129 PMC10557377

[16] W. H. McFadden , D. A. Garteiz , and E. G. Siegmund , “Thermospray Collisionally Induced Dissociation With Single and Multiple Mass Analyzers,” Journal of Chromatography 394, no. 1 (1987): 101–108. 3036899 10.1016/s0021-9673(01)94163-9

[17] R. D. Smith , J. A. Loa , C. J. Barinaga , C. G. Edmonds , and H. R. Udseth , “Collisional Activation and Collision‐Activated Dissociation of Large Multiply Charged Polypeptides and Proteins Produced by Electrospray Ionization,” Journal of the American Society for Mass Spectrometry 1, no. 1 (1990): 53–65. 24248611 10.1016/1044-0305(90)80006-9

[18] S. A. Carr , M. J. Huddleston , and M. F. Bean , “Selective Identification and Differentiation of N‐ and O‐Linked Oligosaccharides in Glycoproteins by Liquid Chromatography‐Mass Spectrometry,” Protein Science 2, no. 2 (1993): 183–196. 7680267 10.1002/pro.5560020207 PMC2142339

[19] A. G. Harrison , “Energy‐Resolved Mass Spectrometry: A Comparison of Quadrupole Cell and Cone‐Voltage Collision‐Induced Dissociation,” Rapid Communications in Mass Spectrometry 13, no. 16 (1999): 1663–1670. 10440985 10.1002/(SICI)1097-0231(19990830)13:16<1663::AID-RCM695>3.0.CO;2-T

[20] M. Hamdan and O. Curcuruto , “Collision‐Induced Dissociation of Some Protonated Peptides With and Without Mass Selection,” Rapid Communications in Mass Spectrometry 8, no. 3 (1994): 274–279. 8167372 10.1002/rcm.1290080310

[21] V. Katta , S. K. Chowdhury , and B. T. Chait , “Use of a Single‐Quadrupole Mass Spectrometer for Collision‐Induced Dissociation Studies of Multiply Charged Peptide Ions Produced by Electrospray Ionization,” Analytical Chemistry 63, no. 2 (1991): 174–178. 1812794 10.1021/ac00002a016

[22] J. A. Loo , C. G. Edmonds , and R. D. Smith , “Tandem Mass Spectrometry of Very Large Molecules: Serum Albumin Sequence Information From Multiply Charged Ions Formed by Electrospray Ionization,” Analytical Chemistry 63, no. 21 (1991): 2488–2499. 1763807 10.1021/ac00021a018

[23] I. X. Peng , J. Shiea , R. R. Ogorzalek Loo , and J. A. Loo , “Electrospray‐Assisted Laser Desorption/Ionization and Tandem Mass Spectrometry of Peptides and Proteins,” Rapid Communications in Mass Spectrometry 21, no. 16 (2007): 2541–2546. 17639579 10.1002/rcm.3154

[24] A. Vellaichamy , J. C. Tran , A. D. Catherman , et al., “Size‐Sorting Combined With Improved Nanocapillary Liquid Chromatography‐Mass Spectrometry for Identification of Intact Proteins up to 80 kDa,” Analytical Chemistry 82, no. 4 (2010): 1234–1244. 20073486 10.1021/ac9021083 PMC2823583

[25] I. A. Hendriks , S. C. Larsen , and M. L. Nielsen , “An Advanced Strategy for Comprehensive Profiling of ADP‐Ribosylation Sites Using Mass Spectrometry‐Based Proteomics,” Molecular & Cellular Proteomics 18, no. 5 (2019): 1010–1026. 30798302 10.1074/mcp.TIR119.001315 PMC6495254

[26] K. Nowak , F. Rosenthal , T. Karlberg , et al., “Engineering Af1521 Improves ADP‐Ribose Binding and Identification of ADP‐Ribosylated Proteins,” Nature Communications 11, no. 1 (2020): 5199. 10.1038/s41467-020-18981-w PMC7566600 33060572

